# Genomic characterization, in vitro, and preclinical evaluation of two microencapsulated lytic phages VB_ST_E15 and VB_ST_SPNIS2 against clinical multidrug-resistant *Salmonella* serovars

**DOI:** 10.1186/s12941-024-00678-3

**Published:** 2024-02-15

**Authors:** Reem A. Youssef, Masarra M. Sakr, Rania I. Shebl, Bishoy T. Saad, Khaled M. Aboshanab

**Affiliations:** 1 Department of Microbiology and Immunology, Faculty of Pharmacy, Ahram Canadian University, Giza, Egypt; 2 Microbiology and Immunology Department, Faculty of Pharmacy, Ain Shams University, African Union Organization St, Abbassia, 11566 Cairo Egypt; 3 Department of Bioinformatics, HITS Solutions Co, Cairo, 11765 Egypt

**Keywords:** *Salmonella*, Multidrug-resistant; Bacteriophage, Microencapsulation, Histopathology, electron microscopy

## Abstract

**Background:**

*Salmonella* infections continue to be one of the essential public health issues threatening millions of people. With the increasing occurrence of resistance against conventionally used antibiotics, the search for alternatives has become crucial. In this study, we aimed to isolate, characterize, and evaluate two lytic bacteriophages against clinically isolated multidrug-resistant (MDR) *Salmonella* serovars.

**Methods:**

Screening for the phage lytic activity was performed using a spot test. Characterization of the isolated phages was done by determining the host range, longevity test, and the effect of temperature, pH, organic solvents, and morphological characterization using a transmission electron microscope. Genomic analysis was performed using Oxford nanopore sequencing. The lytic activities of the free phage lysates and formulated phage as microencapsulated were evaluated both in vitro and in vivo.

**Results:**

Two phages (VB_ST_E15 and VB_ST_SPNIS2) were successfully isolated and showed lytic strong activities against MDR *Salmonella* (*S.)* Typhimurium ATCC 14,028, *S.* Paratyphi A, and *S.* Typhi. The two phages survived at the tested temperatures, maintained their infectivity for 90 days, and retained their activity until 60 °C with thermal inactivation at 65 °C. They were lytic at a pH range from 3 to 11 but lost their activities at extremely acidic or alkaline pH. The phages could withstand the organic solvents but were completely inactivated by 100% ethanol. Both phages were classified under the order *Caudoviricetes*, and Genus: *Uetakevirus*. Their genomic sequences were assembled, annotated, and submitted to the NCBI GenBank database (OR757455 and OR757456). The preclinical evaluation using the murine animal model revealed that the two-phage cocktail managed MDR *Salmonella* infection as evidenced by the reduction in the bacterial burden, increased animal weight, and histopathological examination.

**Conclusion:**

The two encapsulated phage formulas could be considered promising candidates for the management of MDR *Salmonella*-associated infections and clinical analysis should be undertaken to evaluate their potential use in humans.

**Supplementary Information:**

The online version contains supplementary material available at 10.1186/s12941-024-00678-3.

https://orchid.org/0000-0002-7608-850X.

## Background

Humans are susceptible to a variety of *Salmonella* infections. Depending on *Salmonella* serotypes, the clinical syndromes associated with its pathogenicity could range from self-limiting gastroenteritis commonly caused by nontyphoidal *Salmonella* (NTS) to potentially fatal typhoidal fever [1]. Compared to typhoidal strains that are human host-specific, NTS exhibits a broad host specificity [2]. In terms of epidemiology, NTS affects the entire world, as opposed to typhoidal *Salmonella*, which is principally endemic in developing nations [3].

NTS is considered a major source of foodborne illness in humans around the world, known as salmonellosis. Globally, NTS causes about 94 million infections and 155,000 fatalities annually [4]. NTS gastroenteritis is usually self-limiting resolving within days but occasionally it leads to invasive diseases like respiratory and urinary tract infections, vasculitis as well as bacteremia [5]. Moreover, invasive NTS infections (iNTS) can be life-threatening in infants and elderly patients with immune deficiency [6]. In 2017, an alarming report estimated that the case-fatality of *Salmonella* reached 14.7% and it was attributed to iNTS sickness, with 535,000 illnesses and 77,500 fatalities worldwide [7]. Additionally, the largest burden of *Salmonella* infections as well as 85.8% of all iNT*S* deaths worldwide were found in Sub-Saharan Africa (SSA) [8].

Typhoid and paratyphoid fevers, also known as enteric fever, are febrile infections attributed to *S.* Typhi and *S.* Paratyphi A, B, and C [9]. In 2017, a total of 14.3 million cases of typhoid and paratyphoid fever were recorded including 135,900 deaths, with 15.8% of death cases in Sub-Saharan Africa [7]. Furthermore, *Salmonella* Typhi infection is a common cause of bloodstream infections (BSI), with several outbreak reports since 2012 [10, 11]. Although antibiotic therapy could be life-saving, the growing bacterial resistance to antibiotics due to antibiotic misuse is an essential issue to be considered especially in the case of multiple drug resistance [5]. The categorization of a bacterial strain as multidrug-resistant (MDR) is based on displaying resistance to three or more antimicrobial drug classes [12].

Consequently, finding an alternative to antibiotic therapy is of great concern. One of these potential alternatives to antibiotics is bacteriophages. Bacteriophages are small viruses harmless to humans, but lethal to their specific bacterial hosts. The therapeutic application of lytic phages as antimicrobial agents is considered an environmentally friendly, cost-effective, and sustainable approach [13]. Moreover, bacteriophages provide advantages over antibiotics in terms of their high specificity, low developmental costs as well as reduced resistance rates [14]. Therefore, the current study aimed at isolation, characterization, in vitro and in vivo evaluation of locally isolated lytic bacteriophages as a free lysate and as formulated one against different serovars of clinical MDR *Salmonella*.

## Materials and methods

### *Salmonella* clinical isolates and antimicrobial susceptibility testing

Among thirty-one *Salmonella* isolates applied for the isolation of bacteriophages, twenty-six isolates were previously identified and susceptibility profiled in our Lab [15]. *S.* Paratyphi A, *S.* Paratyphi B, *S.* Paratyphi C clinical isolates, and a standard strain *S.* Typhimurium ATCC 14,028, were supplied by the Bacterial Bank of Animal Health Research Institute (AHRI, Egypt). Moreover, *S.* Typhi clinical isolate was kindly provided by the Central laboratories of the Ministry of Health and Health Insurance, Egypt (Table 1). Bacterial isolates were serologically identified at AHRI according to the White-Kauffman-Le Minor scheme, as reported by the Word Health Organization (WHO) Collaborating Centre for Reference and Research on *Salmonella (*WHOCC-Salm) [16] and they were also assessed for their antibiotic susceptibility pattern according to CLSI guidelines using the Kirby-Bauer disk diffusion [17]. MDR was determined as previously described [12].

**Table 1:** *Salmonella* serovars applied in bacteriophage isolation

Strain no.	Salmonella serovars	Source
1	*S.* Typhimurium ATCC 14,028	AHRI
2	*S.* Typhimurium 1	Our previous study [15]
3	*S.* Typhimurium 2	
4	*S.* Typhimurium 3	
5	*S.* Typhimurium 4	
6	*S.* Typhimurium 5	
7	*S.* Typhimurium 6	
8	*S.* Anatum 1	
9	*S.* Anatum 2	
10	*S.* Hull	
11	*S.* Tesive 1	
12	*S.* Tesive 2	
13	*S.* Tesive 3	
14	*S.* Blegdam	
15	*S.* Lumberhurst 1	
16	*S.* Lumberhurst 2	
17	*S.* Lumberhurst 3	
18	*S.* Lumberhurst 4	
19	*S.* Entertidis 1	
20	S. Entertidis 2	
21	S. Entertidis 3	
22	*S.* Tumodi	
23	*S.* Taksony	
24	*S.* Dublin	
25	*S.* Agama	
26	*S.* Poona	
27	*S. Butontan*	
28	*S.* Paratyphi A	AHRI
29	*S. Paratyphi C*	
30	*S.* Paratyphi B	
31	*S.* Typhi	Central laboratories of the Ministry of Health and Health Insurance

AHRI; Animal Health Research Institute, Giza, Egypt

### Isolation of *Salmonella*-specific bacteriophages

The bacterial host employed in phage isolation was *S.* Typhimurium ATCC 14,028, the isolate was cultured overnight in Tryptic Soy Broth (TSB), and the bacterial count was obtained by dilution in TSB to match the turbidity of 0.5 McFarland standard (10^8^ colony forming unit (CFU)/mL). Samples employed for bacteriophage isolation were collected from different poultry markets in Cairo and Giza governorates as raw chicken rinse samples. Samples were filtered using filter paper to remove suspended particulates [18]. A fresh rinse of raw chicken (5 mL) was incubated with 5 mL of a mixture of the previously prepared bacterial isolate in addition to 50 mL of double-strength TSB (supplemented with 10 mM CaCl_2_ and 1 M MgSO_4_). The mix was incubated overnight at 37 °C (200 rpm), followed by centrifugation at 2817 x g for 20 min using 1010 Centrifuge, Centurion Scientific ltd, UK. The supernatant was shaken with chloroform (1% v/v) for 30 min at room temperature to kill the bacterial cells followed by getting rid of the bacterial cell remnants by centrifugation at 2817 x g for 10 min. The obtained phage lysate was kept at 4 °C [19].

### Screening for the phage lytic activity against MDR *Salmonella*

The phage lysate was screened for its lytic activity against MDR *Salmonella* serovars using a spot test [19]. The plaque assay was carried out to settle the titer of the isolated phage(s) in their initial lysate using the standard double agar overlay (DAO) method [20, 21]. Ten-fold serial dilution of the phage lysate in saline-magnesium (SM) buffer: NaCl, 5.8 g, 100 mM. MgSO4•7H2O, 2 g, 8 mM. Tris-Cl (1 M, pH 7.5), 50 ml, 50 mM. H2O, to 1 L. (Biodiagnostics ®, Cairo, Egypt) was done. Each phage dilution was mixed with an equal volume of the bacterial host followed by adding this mixture to a 3 mL soft overlay composed of double-strength TSB and agar at a concentration of 0.75 g/100 mL [22]. Each mixture was then poured and evenly distributed over a layer of TSA previously prepared. The plates were left to completely solidify undisturbed and were incubated in an upright position at 28 °C overnight. The plaques were counted and the phage titer was calculated as previously reported [23].

### Purification of the isolated bacteriophages

To ensure the purity of the suspended phage lysate, a sterile spatula was used to pick out a single well-defined plaque and suspend it in SM buffer. It was then left for 2 h and then the obtained suspension was incubated overnight with fresh bacterial host, centrifuged, treated with chloroform, and kept at 4 °C [19].

### Phages propagation

Phage cocktail propagation was performed by repeating the previously described method for phage isolation three times using aliquots of the phage lysate as an alternative to the chicken rinse samples [19, 24].

### Characterization of the isolated bacteriophages showing lytic activity against MDR *Salmonella* isolates

Characterization was assessed using a spot test which showed either a positive (clear spot) or negative result (no spot) indicating that the phage was either active or inactive after being challenged using different *Salmonella* isolates and also at different pH values, temperatures, or organic solvents.

### Host range

The host range of the isolated phage cocktail was evaluated using a spot test against 31 MDR *Salmonella* isolates [25]. For each tested bacterial isolate, a plate with a TSA base layer was overlaid with 3 mL of double-strength TSB agar inoculated with the tested MDR *Salmonella* isolate. Then, each plate was spotted with 15 µL of the phage lysate, overnight incubated followed by visual examination. The phage lytic effect was detected by the presence of an inhibition zone in the area of the applied spot surrounded by a well-grown confluent sheet of bacterial growth.

### Longevity test

Aliquots of the previously prepared bacteriophage lysate were maintained at 4, 37, and − 80 °C. The phages were examined for their lytic activity at 1, 2, 3, 4, 5, 6, 7, 15, 30, 60, and 90-day intervals using spot test [19].

### Thermal stability

Aliquots of the phage lysates were incubated at different temperature ranges between 30 and 65 °C (5-degree intervals) for 1 h followed by an examination of their lytic activity using spot test. The thermal inactivation point was considered as the temperature at which a complete loss of the phage lytic activity was recorded [25].

### pH stability

The phage lysate cocktail was mixed with an equal volume of TSB at different pH ranges between 1 and 13. The prepared suspensions were left for 1 h at room temperature then the phage lytic activities were observed using spot test [26].

### Sensitivity to organic solvents

The phage lysates were treated with different concentrations of ethanol, isopropyl alcohol, and chloroform (10, 30, 50, and 100% v/v), incubated at room temperature for 1 h, and evaluated for their lytic activity using spot test according to the studies conducted by Abd-Allah et al. and Oduor et al. [19, 27].

### Morphology of the isolated bacteriophages

Morphological characterization of the isolated phages against MDR *Salmonella* was performed using a transmission electron microscope (TEM) via preparation of a high-titer phage lysate previously prepared using 2 to 3 successive propagations. Purification was performed by centrifugation for 25 min at 7826 x g twice, followed by syringe filtration (0.22 μm). Samples were sent to NanoTech for Photo Electronics Co. Giza, Egypt, for characterization by TEM. They were prepared following the procedure described by Kalatzis et al. [28] and examined using a transmission electron microscope (JEOL_JEM_2100 Electron Microscope Siemens & Halske, Germany) [25, 28].

### Molecular analysis of the isolated bacteriophages

#### Extraction of nucleic acid and genomic sequencing

The genomic DNA of the purified phage lysate was extracted using QIAamp® DNA Minikit (QIAGEN, Hilden, Germany) according to the manufacturer’s instructions. Ensuring the quality and quantity of the extracted DNA was carried out as recommended by the specifications outlined in the kit’s user guide.

#### Genomic sequencing of phage genome

The phage lysate was sequenced in an Illumina MiSeq instrument (Illumina, La Jolla, CA, USA), at Children Cancer Hospital 57,357, Cairo, Egypt, and the library was set up by the Nextera XT DNA Library preparation kit (San Diego, CA, USA). The obtained contigs were assembled using the StadenPackage software v2. Further confirmation of certain gaps has been performed using the Oxford Nanopore Sequencing [29] which was carried out at HITS Solutions, Co, (https://www.hitssolutions.com/), Cairo, Egypt.

#### Library preparation and Oxford nanopore sequencing

Preparation of the genomic library was carried out using a Rapid Barcoding Kit (SQK-RBK004; Oxford Science Park, OX4 4DQ, UK) according to the manufacturer’s protocol. The quality of the Fastq reads was assessed using Fastqc. Subsequently, reads that were of low quality or insufficient length were removed using the NanoFilt tool https://github.com/wdecoster/nanofilt (accessed on October, 2023) [30]. Adapter sequences present in the reads were then eliminated using Porechop_ABI https://github.com/bonsai-team/Porechop_ABI (accessed on October, 2023) [31]. The filtered reads were then subjected to Denovo assembly and polishing using Flye https://github.com/fenderglass/Flye (accessed on October, 2023) and Medaka https://github.com/nanoporetech/medaka (accessed in October, 2023), respectively. The final consensus sequence was analyzed using PATRIC BRC [32] https://www.bv-brc.org/ (accessed on 23 October 2023) and analyzed using RAST algorithm [33]. The final assembled consensus sequence of each phage genome was annotated [34] and submitted to the NCBI GenBank database under the accession code, OR757455 and OR757456. The BLAST Ring Image Generator (BRIG) tool v0.95 (https://sourceforge.net/projects/brig/ (accessed on 25 October 2023) was employed to create the circular image [35].

### Microencapsulation of *Salmonella* phages cocktail using freeze drying

Freeze drying was applied as a method for microencapsulation of the isolated phages using Whey protein isolate (WPI) protein (Sigma Aldrich, Saint Louis, MO, USA) which was prepared as previously reported [36]. Trehalose dihydrate (Advent Chembio Pvt Ltd, India) was immediately incorporated into the whey protein solution at a ratio of 1:3 according to the optimized formulation as demonstrated by Petsong et al. [37] to achieve 10% (w/v) total solid. Phages lysate (10^9^ PFU/mL) was added to the mixture to obtain 10% (v/v). The mixture was held at -80˚C for 12 h. The frozen mixture was dried at -50˚C using a laboratory scale freeze-dryer (CoolSafe 55, ScanLaf A/S, Lynge, Denmark) under vacuum (Welch, 8912 Vacuum pump, Gardner Denver Thomas, Inc, Welch Vacuum Technology Niles, IL, USA) at approximately 30 pounds per square inch (psi) for 48 h. Dry lyophilized powder was obtained (10 ± 0.05 g) and employed for the assessment of the phage titer [37].

### In vitro antimicrobial activity of microencapsulated phages

Freeze-dried phage powder (1 g) was resuspended in SM buffer (10 mL) and incubated for 1 h at room temperature while shaking (220 rpm) (ThermoStableTM IS-30 model, DAIHAN Scientific, Korea). Plaque-forming units were determined to evaluate the ability of the phage cocktail to retain its lytic activity post-encapsulation. The plaque forming units were evaluated against the three selected MDR bacterial hosts. The encapsulation efficiency (EE) was estimated using the identified phage titer originally applied for phage encapsulation (TP) compared to the phage titer recovered from the dried powder (RP), using the following formula: EE (%) = RP/TP × 100 [37].

### In vivo antimicrobial potential of microencapsulated phages

#### Laboratory animals and experimental design

Sixty-five male white albino mice aged 6 weeks and weighing between 100 and 125 g were used as animal models throughout the experiment. Animals were kept in open cages, and given antibiotics-free food as previously reported [38]. Animals were maintained on an alternate 12 h light-dark cycle, with a constant temperature of 25 °C controlled by air conditioning at the Animal House Facility (Faculty of Pharmacy, Ahram Canadian University, Giza, Egypt). Dealing with animals was carried out according to the regulations of the animal care and use committee of the Faculty of Pharmacy, Ain Shams University (ACUC-FP-ASU) as recommended by the National Regulations on Animal Welfare and the Institutional Animal Ethical Committee. Animals were categorized into thirteen groups (5 mice / each) as previously described [38]. Fecal samples from 5 mice belonging to different groups were obtained before the beginning of the experiment and checked for the absence of *Salmonella* infection. Treatment was done by either the free phage lysate cocktail, the formulated microencapsulated phage cocktail, or by using the vehicle only (10% whey protein and trehalose in ratio 3:1 dissolved in sterile distilled water containing 10% TSB) as a control (Table 2). Each mouse was infected orally with a single dose (0.2 mL) of *Salmonella* suspension of the selected serovar (1.5 × 10^8^ CFU/mL). On the third day post infection, infected mice were treated with either the free form of the phage cocktail or the microencapsulated phage daily for 9 days (1 × 10^8^ PFU/mL, 0.2 mL/dose). On the 12th day, mice were weighed prior to slaughtering, and a part of the liver of each mouse in each group was aseptically removed and homogenized for determination of the bacterial viable count [38]. All internal mice organs were preserved in 10% formalin for further histopathological investigations.

**Table 2:** Categorization of animals and the description of each group

Group number	Description
Group I	uninfected, untreated
Group II	infected by *S.* Typhimurium ATCC 14,028, untreated
Group III	infected by S. Paratyphi A, untreated
Group IV	infected by S. Typhi, untreated
Group V	infected by *S.* Typhimurium ATCC 14,028, treated by free form
Group VI	: infected by S. Paratyphi A, treated by free form
Group VII	infected by S. Typhi, treated by free form
Group VIII	infected by *S.* Typhimurium ATCC 14,028, treated by microencapsulated form
Group IX	infected by S. Paratyphi A, treated by microencapsulated form
Group X	infected by S. Typhi, treated by microencapsulated form
Group XI	infected by *S.* Typhimurium ATCC 14,028, treated by vehicle
Group XII	infected by S. Paratyphi A, treated by vehicle
Group XIII	infected by S. Typhi, treated by vehicle

#### Histopathology

For the histopathological analysis, the liver, spleen, intestine, and mesenteric lymph nodes (MLN) were fixed in 10% neutral-buffer formalin [39]. The samples were obtained from each of the dissected organs, washed with tap water, and dehydrated for 30 min using diluted ethanol. The samples were incubated in a 1:1 mixture of ethanol and xylene for 30 min, washed twice using xylene for 1 h, and moved to xylene and paraffin mixture for 30 min. Transverse Sect. (5 mm) were dewaxed at 60 °C, immersed in xylene for 1 h, rehydrated using a series of ethanol for 2 min, and washed using tap water. Sections were stained using hematoxylin and eosin, mounted using Entellan embedding agent, and examined [40] for histopathological abnormalities at the Pathology laboratory (AHRI, Giza, Egypt) in a blind manner.

#### Bacterial viable count

Dissected livers were weighed and homogenized in SM buffer using a hand-held tissue grinder. Ten-fold serial dilutions of homogenized liver were prepared and plated onto *Salmonella*-*Shigella* agar plates and incubated at 37 °C for 24 h. Bacterial counts were measured per one gram of the homogenized tissue.

### Statistical analysis

Data were analyzed by one-way analysis of variance (ANOVA) using Graph pad Instat-3 software (Graph Pad Software Inc., USA), based on the normality of the data that was assessed using the Kolmogorov-Smirnov and Shapiro-Wilk tests (Table S1, Figures S1 and S2) and the homogeneity of variances for log bacterial count that was tested using Levene’s test (Tables S3). Results were considered significant at P values less than 0.001 (Table S3) and were presented as means ± standard deviation.

## Results

### *Salmonella* clinical isolates and antimicrobial susceptibility testing

*Salmonella* clinical isolates were found to be 100% MDR. Consequently, they were used as bacterial hosts during the period of the study.

### Plaque assay

**Fig. 1:**
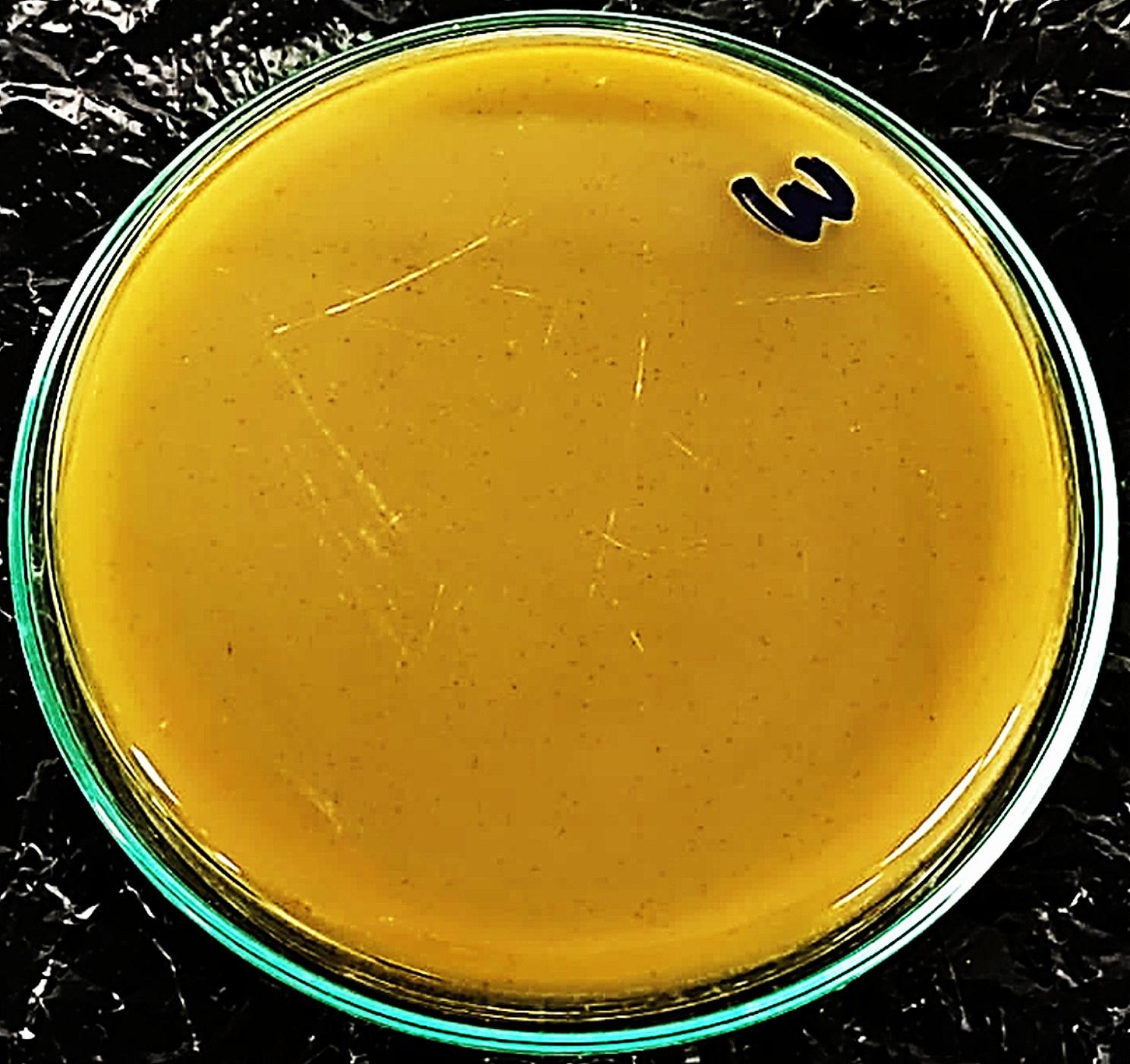
Morphological appearance of plaques induced post exposure to the isolated phage cocktail. Plaques were clear, circular, and very small in size (1–2 mm diameter)

The initial phage titer of the isolated phage cocktail was 1.3 × 10^8^ PFU/mL. The plaques formed by the phage cocktail appeared very small in size (1–2 mm), circular and clear as shown in Fig. 1.

### Characterization of the isolated bacteriophages

#### Host range

Isolated phages showed clear lytic spots against three MDR *Salmonella* isolates, *S.* Typhimurium ATCC 14,028, *S.* Paratyphi A, and *S.* Typhi.

#### Longevity test

Results revealed that the isolated phages survived at all the tested storage temperatures of 4, 37, and − 80 °C. maintaining their infectivity (as indicated by positive spot test) for up to 90 days.

#### Thermal stability

Recorded data showed that the isolated phages retained their activity at all the tested temperatures until 60 °C displayed by forming a clear zone in the spot test while when incubated at 65 °C for 1 h, the recorded lytic activity was lost as indicated by spot test. Thus, the thermal inactivation point was considered 65 °C.

#### pH stability

Isolated phages succeeded in producing clear lytic spots at a pH range 3 to 11. However, at pH 1, 2, 12, 13, no spot was observed indicating loss of phage lytic activities.

#### Sensitivity to organic solvents

Interestingly, the isolated phages withstand the presence of the organic solvents at all the tested conditions. On the other side, they were completely inactivated in the presence of 100% v/v ethanol.

#### Transmission electron microscope (TEM)

**Fig. 2:**
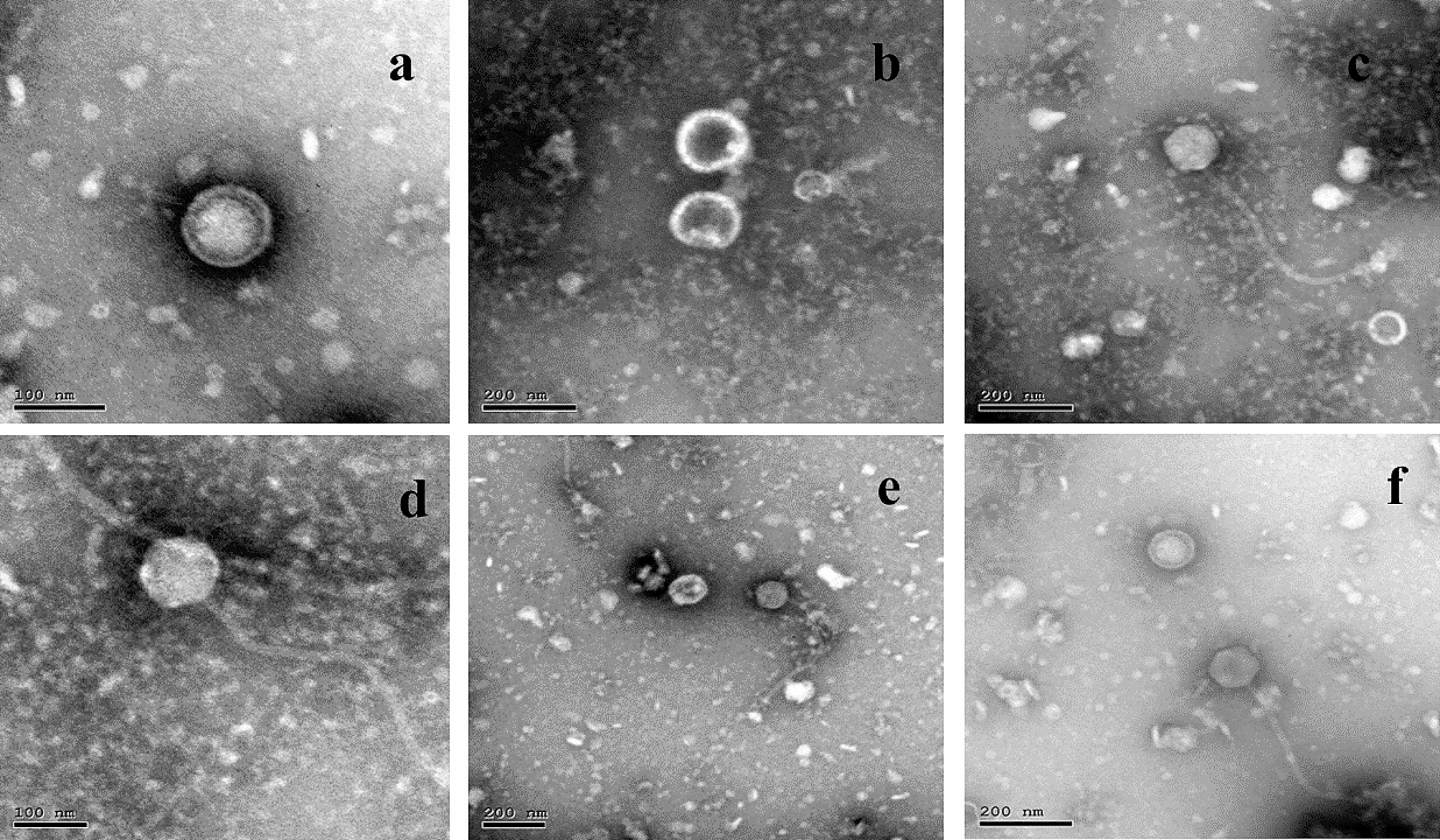
TEM images for the isolated bacteriophages showing **a** & **b**: *Salmonella* phage VB_ST_E15, **c** & **d**: *Salmonella* phage VB_ST_SPNIS2, **e** & **f**: *Salmonella* phage cocktail, Scale bar represents 100, 200 nm

The captured images using the TEM, Fig. 2, suggested the presence of *Salmonella* phage VB_ST_E15, where it appeared as a tailed phage with about 17 nm length, with non-contractile tail, 6 prominent tail spikes, and a head of about 70 nm in diameter. Results also suggested the isolation of *Salmonella* phage VB_ST_SPNIS2 where it appeared as a tailed phage with about 363 nm length, non-contractile tail, 6 prominent tail spikes, and an icosahedral head of about 102 nm in diameter. Both viruses were classified under the order *Caudoviricetes*, and genus *Uetakevirus* (viruses; *Duplodnaviria; Heunggongvirae; Uroviricota; Caudoviricetes; Uetakevirus*). These findings corresponded to the latest rules compiled by the International Committee on Taxonomy of Viruses (https://ictv.global) [41].

### Genomic sequencing of *Salmonella* phage VB_ST_E15 and VB_ST_SPNIS2

**Fig. 3:**
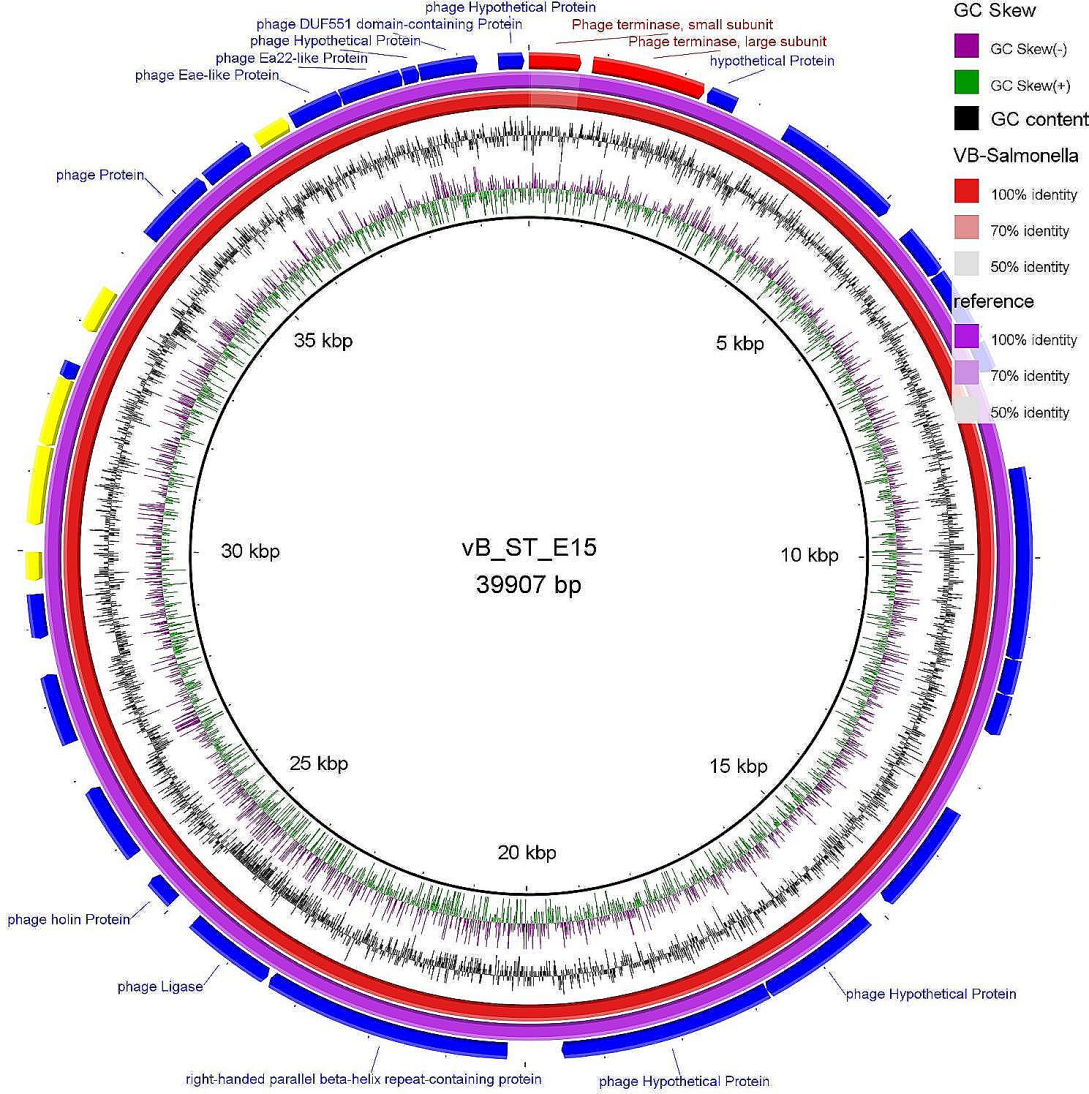
Circular genome map of VB_ST_E15 (purple ring; NCBI accession code, OR757455) and the reference phage (red ring; Enterobacteria phage VB_ST_E15 ; NCBI accession code, NC_004775.2). The color coding of genes indicates the functional categories of putative proteins: phage and hypothetical proteins (blue), terminase protein (red); phage regulatory proteins (yellow). The creation of the circular image was performed using the BLAST Ring Image Generator (BRIG) tool v0.95 (https://sourceforge.net/projects/brig/, accessed on 12 December 2023)

**Fig. 4:**
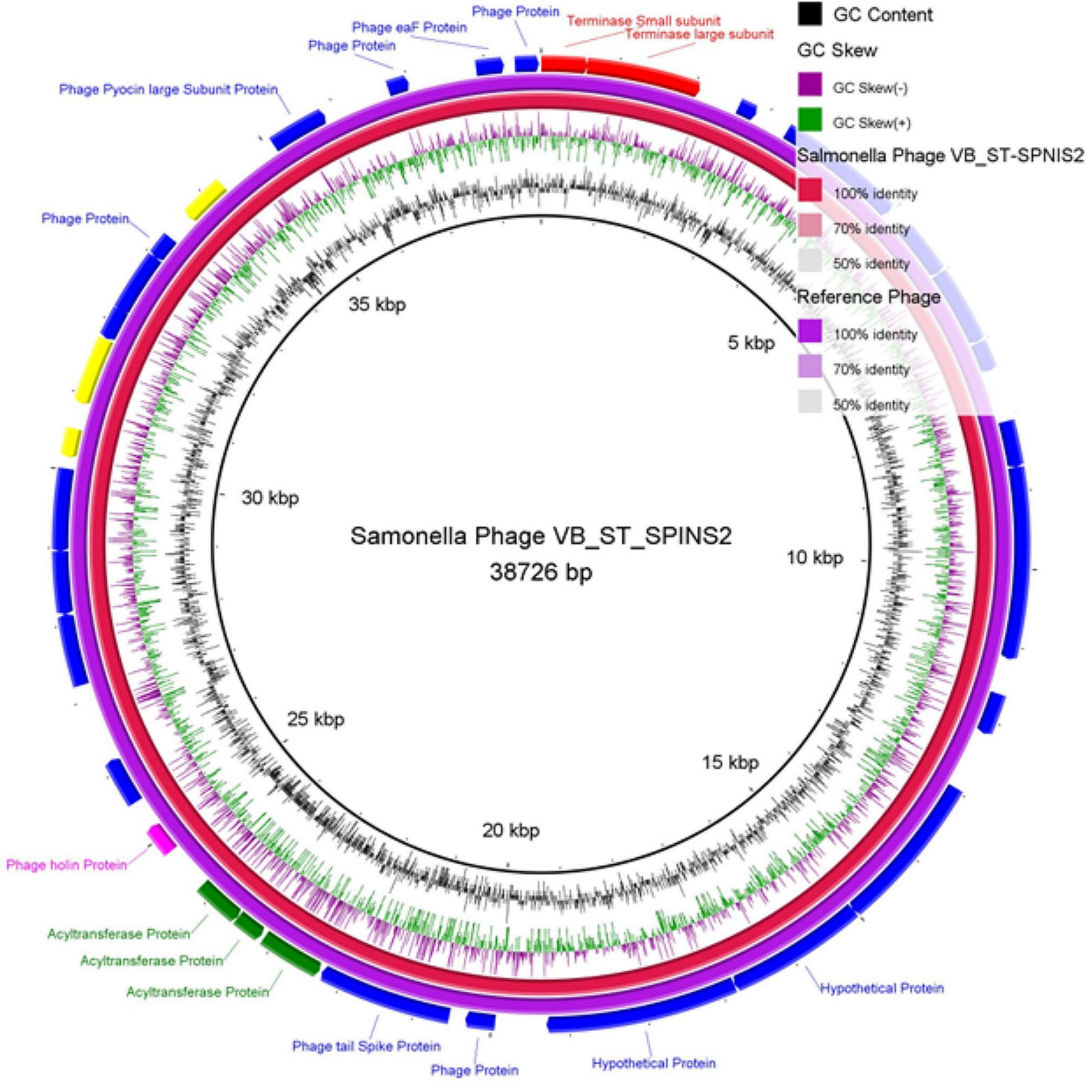
Circular genome map of VB_ST_SPNIS2 (purple ring; NCBI accession code, OR757456) and the reference phage (red ring; Salmonella phage SPC32H; NCBI accession code, KC911856.1). The color coding of genes indicates the functional categories of putative proteins: phage and hypothetical proteins (blue), terminase protein (red); phage regulatory proteins (yellow). The creation of the circular image was performed using the BLAST Ring Image Generator (BRIG) tool v0.95 (https://sourceforge.net/projects/brig/, accessed on 12 December 2023)

The genomic sequence of VB_ST_E15 (32 open reading frames (ORFs) and VB_ST_SPNIS2 (35 ORFs) phages have been assembled, annotated, and deposited in the NCBI GenBank database (accession codes, OR757455 and OR757456, respectively). The number, and positions of the ORFs of each phage are displayed in Tables S4 and S5. BLASTn alignment analysis showed that both phages are taxonomically classified as: viruses; *Duplodnaviria; Heunggongvirae; Uroviricota; Caudoviricetes; Uetakevirus*, with an alignment score > 200 identity. The circular genome maps and the annotated ORFs of VB_ST_E15 and VB_ST_SPNIS2 phages are depicted in Figs. 3 and 4, respectively.

### In vitro antimicrobial activity of microencapsulated phages

#### Microencapsulation efficiency

The microencapsulation efficiency (EE) was estimated to be 91.9% using the known total phage titer initially applied for phage microencapsulation (TP) and the phage titer recovered post encapsulation (RP).

### In vivo antimicrobial potential of microencapsulated phages

#### Histopathology

**Fig. 5:**
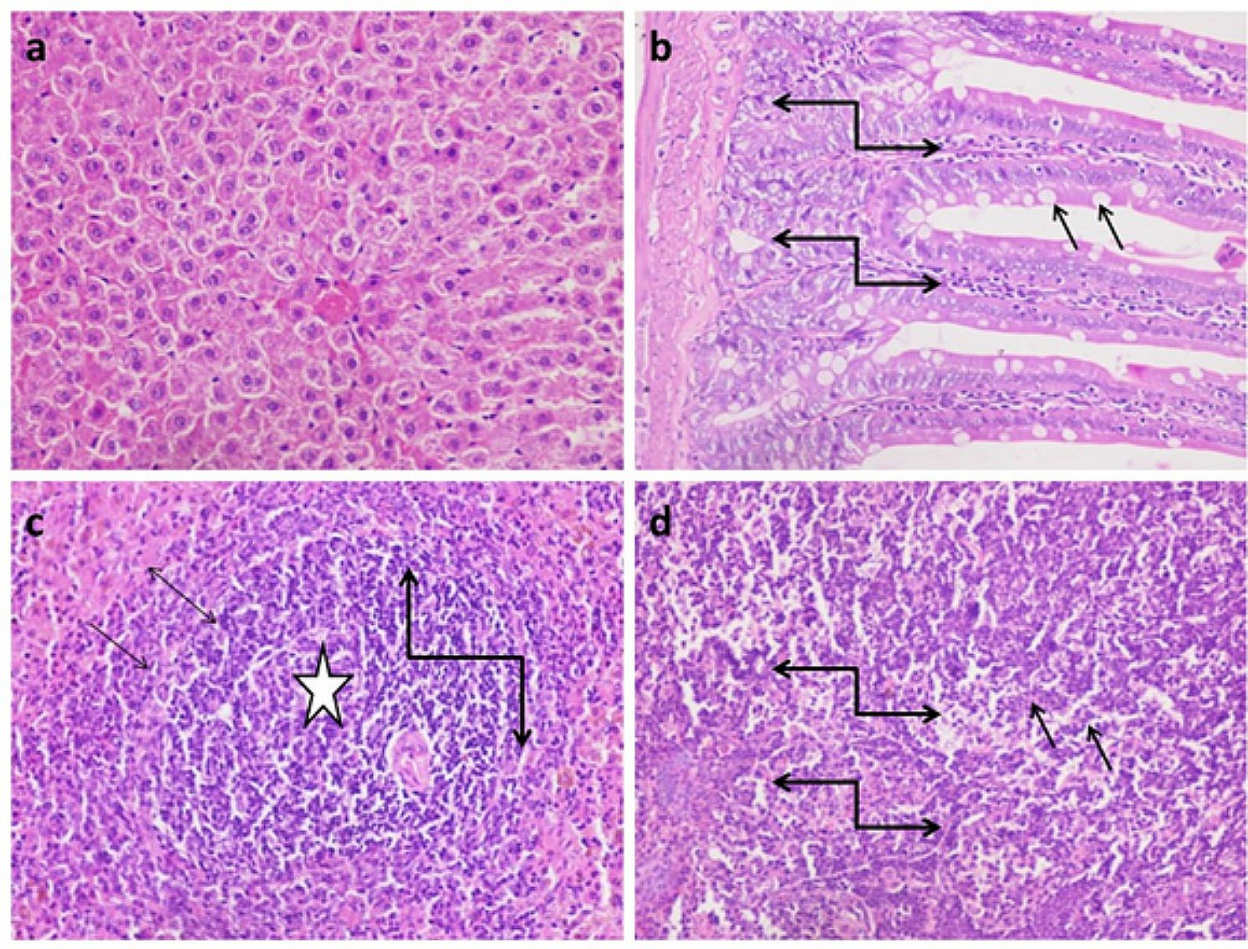
Histopathological Examination of Liver. Group I (uninfected & untreated), **a**: Hepatic parenchyma with normal hepatic architectures, **b**: Intestinal mucosa revealing normal criteria (intestinal villi, goblet cells, arrow and intestinal crypts, truncated arrow), with normal essential cells of lymphocytes and macrophages, **c**: Spleen revealing dark zone (truncated arrow), minute size light zone (asterisk), mantle zone (arrow) and moderate size marginal zone (double headed arrow), **d**: MLN with lymphocytes ( without germinal nor secondary follicle formation; inactive state) embedded in fine reticular stroma and well detected medullary sinuses. (H&E X 400)

**Fig. 6:**
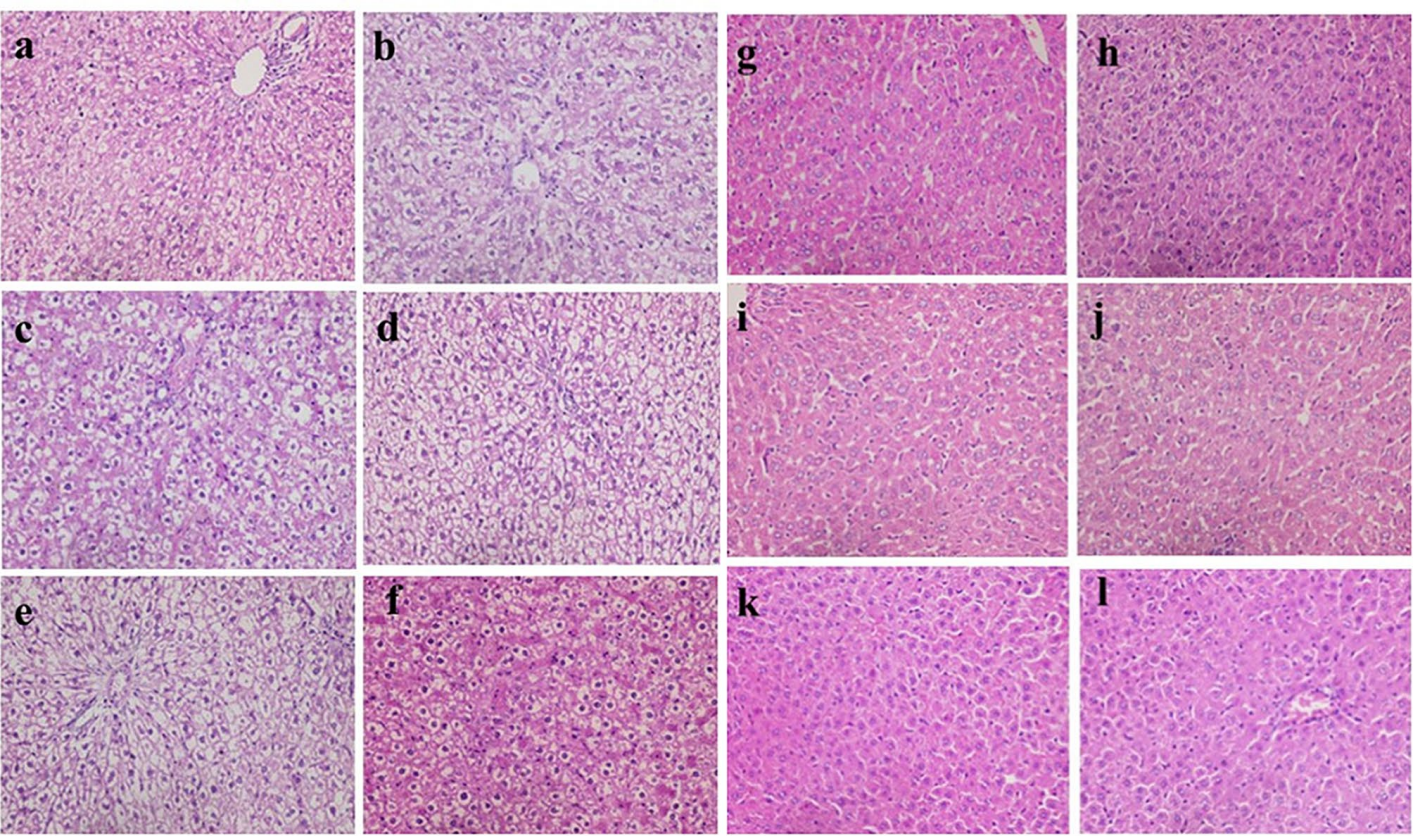
Histopathological examination of liver cells. Fig **a**–**f**: Liver cells with severe degree of hydropic degeneration in all investigated groups. **a**: infected by S. Typhimurium ATCC 14,028, untreated, **b**: infected by S. Paratyphi A, untreated, **c**: infected by S. Typhi, untreated, **d**: infected by S. Typhimurium ATCC 14,028, treated by vehicle, **e**: infected by S. Paratyphi A, treated by vehicle, & **f**: infected by S. Typhi, treated by vehicle. Fig **g**–**l**: Liver cells showing mild dilation of hepatic sinusoids in all groups, some revealing adenoid pattern (**g**, **i** & **j**). Noticeable activation of Ito cells could be detected in all groups. While necrosis of some individual hepatocytes could be demonstrated in other groups (**g**, **i** & **j**) **g**: infected by S. Typhimurium ATCC 14,028, treated by free form, **h**: infected by S. Paratyphi A, treated by free form, **i**: infected by S. Typhi, treated by free form, **j**: infected by S. Typhimurium ATCC 14,028, treated by microencapsulated form, **k**: infected by S. Paratyphi A, treated by microencapsulated form, & **l**: infected by S. Typhi, treated by microencapsulated form (H&E × 400)

**Fig. 7:**
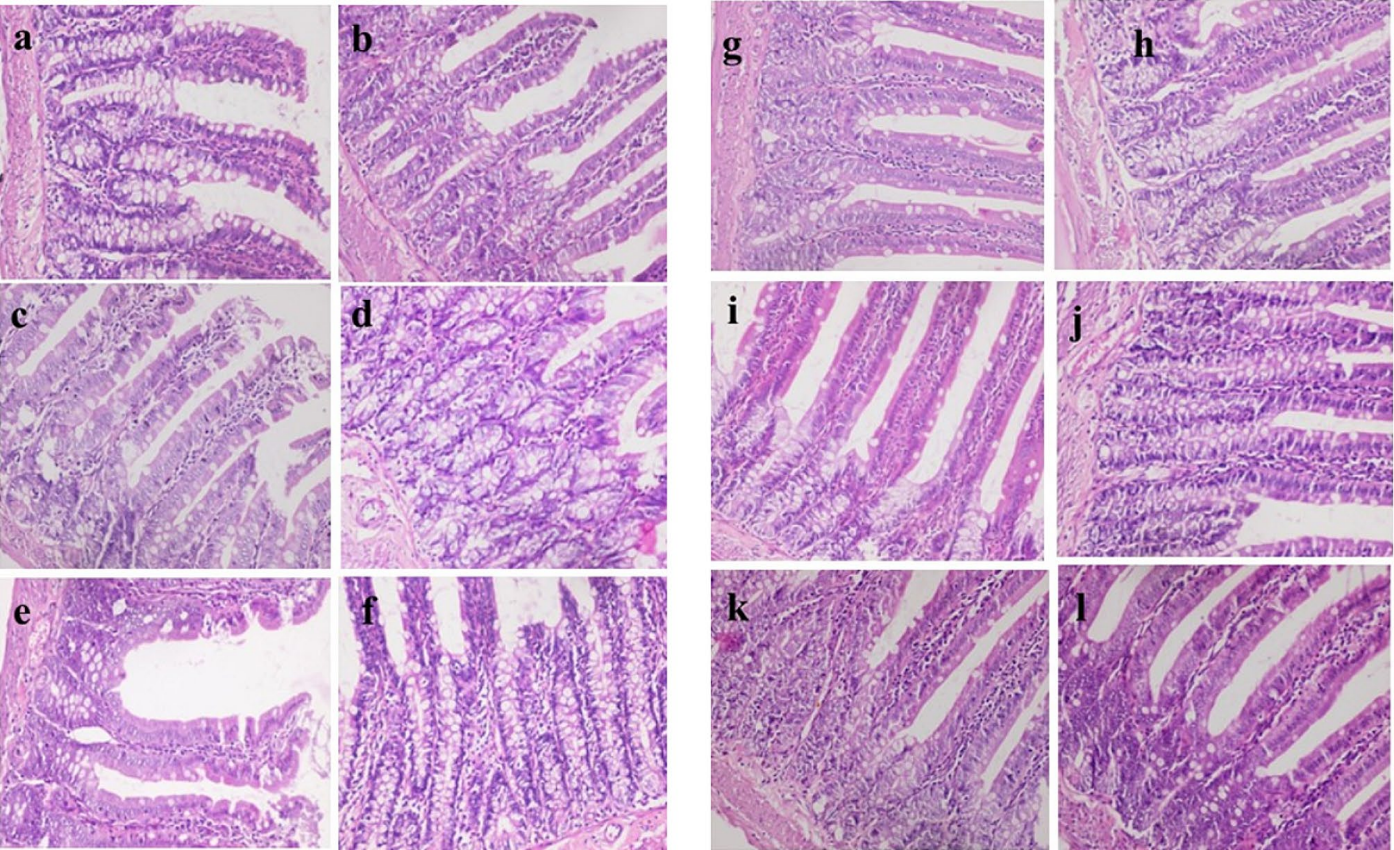
Histopathological Examination of intestinal mucosa. **a**: infected by S. Typhimurium ATCC 14,028, untreated, **b**: infected by S. Paratyphi A, untreated, **c** infected by S. Typhi, untreated, **d**: infected by S. Typhimurium ATCC 14,028, treated by vehicle, **e** infected by S. Paratyphi A, treated by vehicle, & **f**: infected by S. Typhi, treated by vehicle. Intestinal mucosa showing marked dys-figuration of intestinal villi in all groups, marked hyperplasia of intestinal crypts in (**a**, **d** & **f**). The lamina propria in **a**, **b**, **c**, **d** & **e** showed significant number of normal essential cells of lymphocytes and macrophages, while marked depletion detected in **f**, **g**, **h**, **i**, **j**, **k** & **l**: Intestinal mucosa of phage treated groups showed normal criteria with some increase in the length of intestinal crypts in (**j**). The lamina propria harbor normal essential cell population of lymphocytes and plasma cells; in all investigated groups (H&E × 400)

**Fig. 8:**
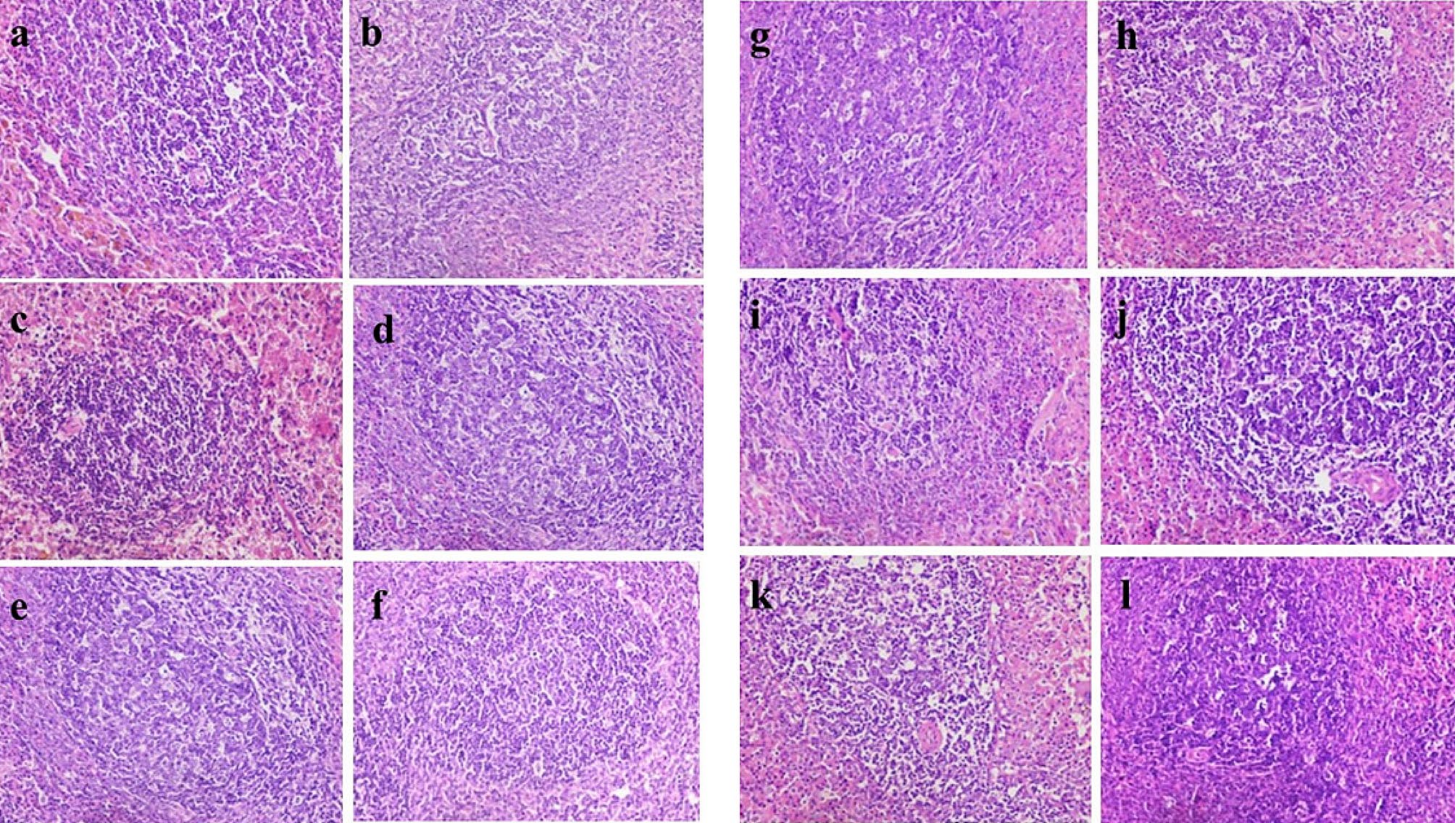
Histopathological Examination of spleen. Spleen showing scant number of tingible bodies in (**b**, **d**, **e** & **f**), while absent in (**b** & **d**), Spleen cells showed well demarcation of dark and marginal zones in all groups with a significant number of tingible bodies in (**g**, **h**, **j**, **k** & **l**) while be absent in (**i**) (H&E × 400)

**Fig. 9:**
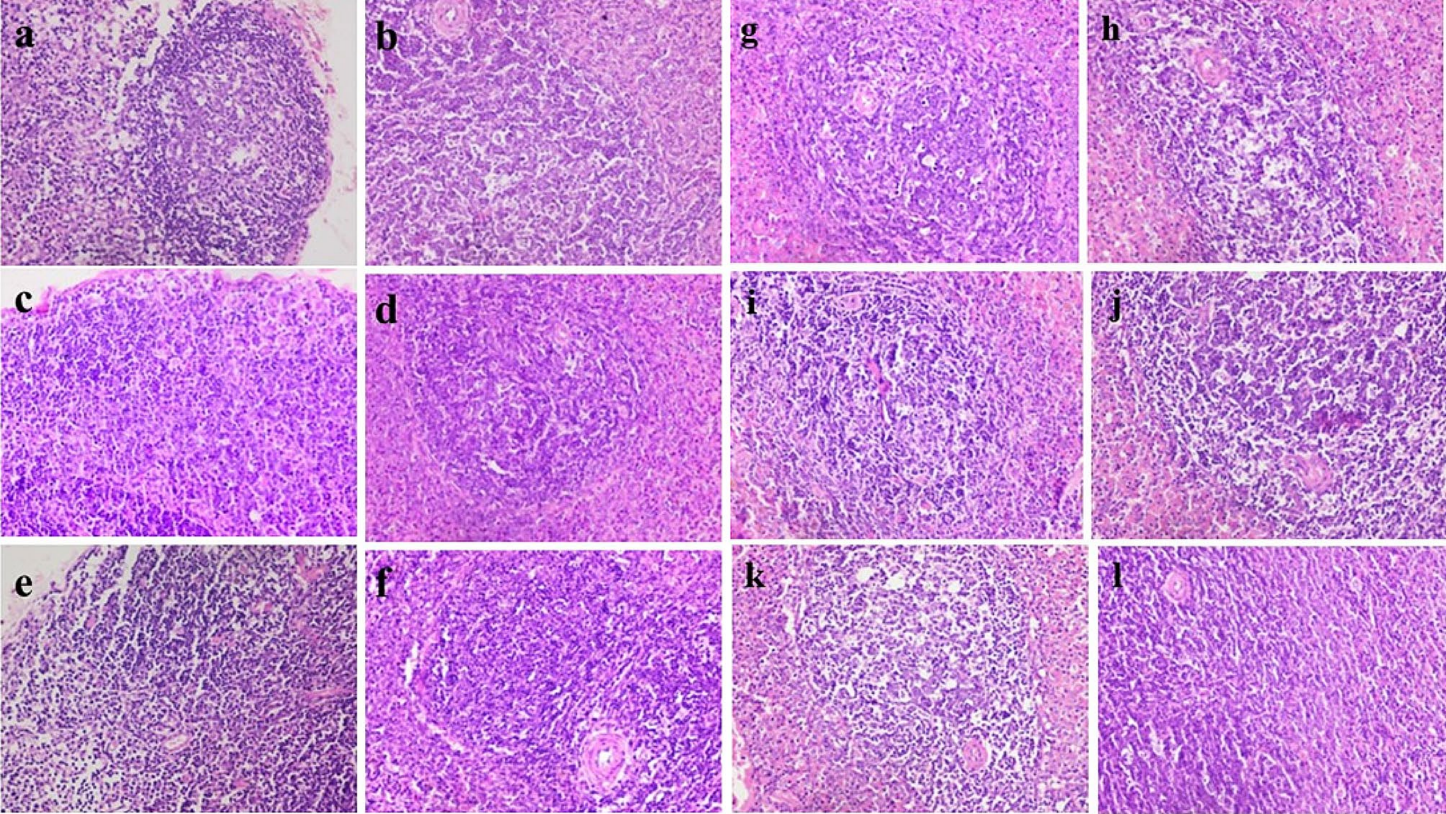
Histopathological examination of mesenteric lymph nodes (MLN). MLN demonstrated variable degrees of lymphoid follicle development: with marked differentiation in (**a**), less degree of differentiation in (**b**, **e** & **f**) with absence of development in (**c** & **e**). Mesenteric LN showing; well development of secondary follicles that could be detected in all groups, with well detected germinal centers in (**g** & **j**). Noticeable tingible body macrophages in (**h** & **i**), with less degree in (**g** & **j**), while scant number in (**i** & **l**) (H&E × 400)

Histopathological examination showed that uninfected & untreated Group I exhibited normal hepatic architectures (Fig. 5a), intestinal mucosa revealed normal appearance of the intestinal villi and goblet cells (arrow) as well as intestinal crypts, (truncated arrow), with normal essential cells of lymphocytes and macrophages (Fig. 5b). Spleen cells showed dark zone (truncated arrow), minute size light zone (asterisk), mantle zone (arrow) and moderate size marginal zone (double headed arrow) (Fig. 5c). MLN appeared with lymphocytes (without germinal nor secondary follicle formation; inactive state) embedded in fine reticular stroma and well detected medullary sinuses (Fig. 5d). Examination of liver cells of infected, untreated and vehicle treated groups showed a severe degree of hydropic degeneration in all investigated groups (Fig. 6a and f). However, liver cells of infected groups that were treated with either the free or microencapsulated phage cocktail showed mild dilation of hepatic sinusoids in all groups, some revealing adenoid pattern. Necrosis of individual hepatocytes was demonstrated as well (Fig. 6g, i and j). Moreover, noticeable activation of Ito cells was detected in all groups. Intestinal mucosa of infected, untreated, and vehicle treated groups showed marked dys-figuration of intestinal villi in all groups with marked hyperplasia of intestinal crypts (Fig. 7a, d and f). The lamina propria in Fig. 7a, b, c, d and e demonstrated a significant number of normal essential cells of lymphocytes and macrophages, while marked depletion was detected in Fig. 7f. On the other side, infected groups that were treated with either the free or microencapsulated phage cocktail revealed normal criteria with some increase in the length of intestinal crypts (Fig. 7j). Also, the lamina propria harbored a normal essential cell population of lymphocytes and plasma cells in all investigated groups. Spleen cells of infected, untreated, and vehicle treated groups demonstrated a scant number of tingible bodies (Fig. 8b, d, e and f), while absent in Fig. 8a and c. However, groups that were infected and treated with either the free or microencapsulated phage cocktail demonstrated well demarcation of dark and marginal zones in all groups with a significant number of tingible bodies (Fig. 8g, h, j, k and l), while absent in Fig. 8i. MLN in infected, untreated and vehicle treated groups showed variable degrees of lymphoid follicle development with marked differentiation (Fig. 9a), less degree of differentiation (Fig. 9b, d and f) with absence of development in Fig. 9c and e. However, infected groups that were treated with either the free or microencapsulated phage cocktail revealed well development of secondary follicles in all groups, with well detected germinal centers (Fig. 9g and j). Noticeable tingible body macrophages were also observed in Fig. 9h and i, with less degree in Fig. 9g and j, while a scant number was demonstrated in Fig. 9k and l.

#### Body weight

**Fig. 10:**
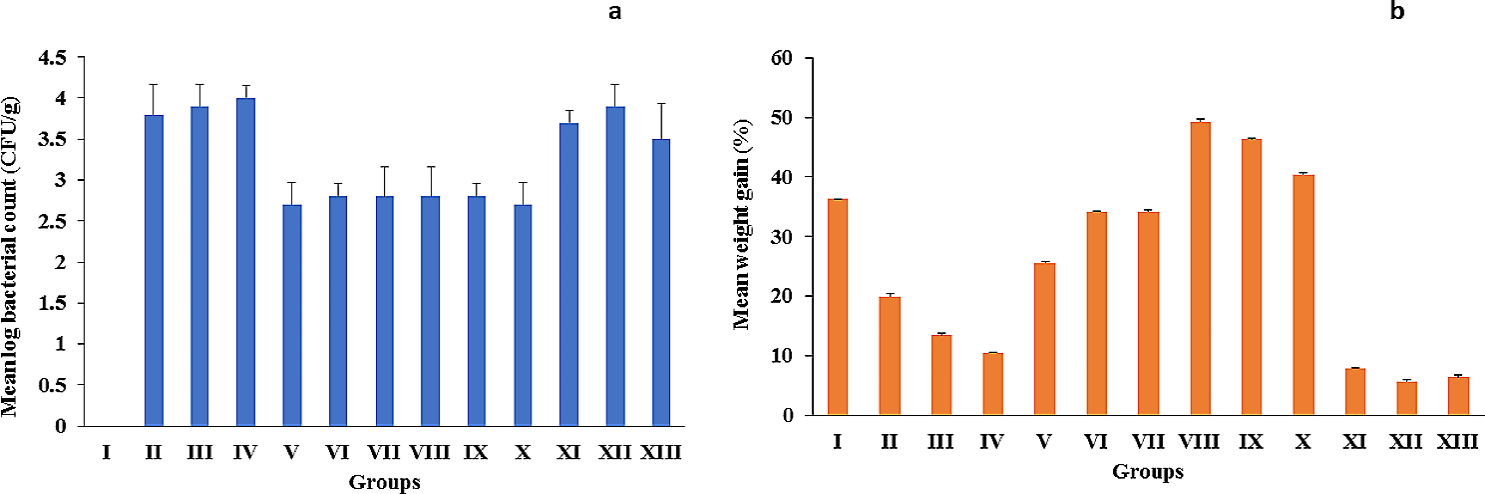
**a**: Mean log bacterial viable count (CFU/g) of *Salmonella* serotypes in the homogenized liver cells among different groups, **b**: Mean weight gain (%) of albino mice in all phage treated and untreated control groups. Data were presented as mean ± SD, *n* = 13

Mice treated with the microencapsulated and the free phage showed a marked increase in the body weight compared to untreated groups as well as vehicle treated groups as shown in Table 3; Fig. 10a.

**Table 3:** Average body weight gain of albino mice and bacterial viable count of *Salmonella* serotypes in liver of animals among the groups

Group	Description	Average weight gain (g)	Average weight gain (%)	Mean log bacterial count in liver ± SD (CFU/g)
Group I	Uninfected, untreated	77 ± 11.5	36.3	0.0 ± 0
Group II	Infected by S. Typhimurium ATCC 14,028, untreated	29.6 ± 3	20	3.8 ± 0.36
Group III	Infected by S. Paratyphi A, untreated	19.5 ± 2.16	13.5	3.9 ± 0.26
Group IV	Infected by S. Typhi, untreated	13.7 ± 0.72	10.5	4 ± 0.15
Group V	Infected by S. Typhimurium ATCC 14,028, phage treated	38.4 ± 3.1	25.6	2.7 ± 0.26
Group VI	Infected by S. Paratyphi A, phage treated	24.3 ± 2.05	19.7	2.8 ± 0.15
Group VII	Infected by S. Typhi, phage treated	53.6 ± 3.3	34.2	2.8 ± 0.36
Group VIII	Infected by S. Typhimurium ATCC 14,028, microencapsulated phage treated	98.7 ± 5.7	49.3	2.8 ± 0.36
Group IX	Infected by S. Paratyphi A, microencapsulated phage treated	91.3 ± 4.5	46.3	2.8 ± 0.15
Group X	Infected by S. Typhi, microencapsulated phage treated	77.7 ± 8.6	40.4	2.7 ± 0.26
Group XI	Infected by S. Typhimurium ATCC 14,028, treated by vehicle	11.5 ± 1.2	7.9	3.7 ± 0.15
Group XII	Infected by S. Paratyphi A, treated by vehicle	7 ± 0.5	5.7	3.9 ± 0.26
Group XIII	Infected by S. Typhi, treated by vehicle	7 ± 0.16	6.4	3.5 ± 0.44

#### Bacterial viable count

Results showed a significant difference between the phage cocktail treated groups and the other groups. The infected untreated and vehicle-treated groups showed the highest count per gram of tissue (mean log bacterial count, 3.5-4) CFU/g, while the lowest count (mean log bacterial count, 2.7–2.8) CFU/g was recorded in the groups treated with the microencapsulated phages cocktail as well as the groups that were treated with the free form of the isolated phage cocktail as shown in Fig. 10b.

## Discussion

Antimicrobial resistance (AMR) among *Salmonella* serovars represents a challenging public threat. This is particularly of great concern in case of resistance to the most important and high-priority antimicrobials, like third-generation cephalosporins and fluoroquinolones [42]. Viruses known as bacteriophages are those that infect only specific bacterial strains. In contrast to antibiotics that harm the natural microbiota and result in subsequent infections, the unique antibacterial activity of bacteriophage therapy is of potential significance [43]. Furthermore, bacteriophages are superior to antibiotics due to their excellent specificity, low developmental costs, and reduced rates of resistance. Thus, the present study aimed to isolate, characterize, and evaluate both in vitro and in vivo the isolated bacteriophages with potential effectiveness against MDR *Salmonella* serovars for their application as eco-friendly biological control agents.

In the current study, the antimicrobial susceptibility pattern for thirty-one *Salmonella* isolates revealed that 100% of the isolates were MDR. This elevated frequency of MDR phenotypes among *Salmonella* isolates highlights the urgent need for strict policies regulating antibiotic misuse in addition to the importance of finding alternative therapies other than antibiotics [44]. Seven raw chicken rinse samples were obtained from different poultry markets in Cairo and Giza governorates and were employed for bacteriophage isolation. The success in yielding these specific phages reflected that poultry is an essential outstanding source for phage isolation against *Salmonella.*

Plaque assay was described as a valuable method for determining the titer as well as the purity of a specific phage lysate [20]. The ideal result of plaque assay is the presence of distinct, unambiguous zones, or plaques, each of which symbolizes an infectious center and is expressed as PFU/mL [45]. If the assay’s outcome results in a single plaque morphology, defined by its size and form indicating the presence of a single pure phage. On the other hand, if it causes distinct forms of plaques, this suggests the presence of many phages where the difference in their morphology could aid in differentiating between the isolated phages [20, 46]. . For instance, clear, transparent plaques are typically associated with lytic (virulent) phages, while turbid, opaque plaques are associated with lysogenic (temperate) phages [47, 48]. Additionally, a small percentage of phages have halos encircling the plaques; these are typically explained by the release of specific enzymes that spread outward from the infectious center. These enzymes most likely exhibit activity against bacterial cell walls and the biofilm that many bacteria create [49, 50]. Moreover, phages may share a similar plaque morphology if they belong to the same order, and family [51]. Also, it was mentioned that similar host ranges and plaque features were shared among isolates with the same morphological type [52]. Upon examination of the two phage plaques in the current study, both plaques had similar plaque morphology as they were very small (1–2 mm), round with a full margin, and crystal-clear zones. That highlights the lytic activity of the isolated phages. Having polyvalent lytic activity against three of the most clinically important isolates, this bacteriophage cocktail was considered promising to continue the study.

The classification of phage particles is preliminary guided by their morphological characteristics. Even though several bacteriophage morphologies have been documented, and despite recent arguments against the traditional phage order requirement [53], Caudoviricetes have been categorized as the order that includes tail-bearing phages. They can be distinguished by their tails, and they are also recognized by their double-stranded DNA and an icosahedral capsid.

The technique employed for the visualization of phage particles involves TEM examination of the negatively-stained phage specimens [54]. TEM analysis was performed on the concentrated and purified phage sample. The phages were classified under the order Caudoviricetes as they had tails. They had icosahedral head and base plates and head-to-tail connectors were absent. VB_E15_SP was characterized by a short tail (17 nm) non-contractile, has 6 prominent tail spikes and a relatively large head (70 nm), while VB_ST_SPNIS2 was characterized by a long non-contractile tail (363 nm), has 6 prominent tail spikes and a large head (102 nm). By comparing these characteristics with those mentioned in (ICTV)-ninth report guidelines [55], it was proposed that the isolated phages VB_E15_SP and VB_ST_SPNIS2 might belong to the order Caudoviricetes. It is essential to point out that genomic characterization is the only acceptable criteria that could be applied to classify the isolated phages, not their morphological features. Accordingly, we performed genomic sequencing for the isolated phages as it is the gold standard for the verification and categorization of the phages. Consequently, the genotypic analysis revealed that phages VB_ST_E15 and VB_ST_SPNIS2 were classified taxonomically as; viruses; *Duplodnaviria; Heunggongvirae; Uroviricota; Caudoviricetes; Uetakevirus*. Moreover, the resulting ORFs of both phages do not show any antibiotic-resistant genes or virulence genes which is considered an additional safety parameter for future therapeutic use of the respective phages.

Microencapsulation is a popular technique for enhancing the stability and bioavailability of bioactive substances. Microencapsulation is applied for the production of microcapsules or microparticles that have a layer of wall materials around their core, which contains the bioactive compounds [56]. The wall of the microcapsule enhances the stability of the encapsulated substances by acting as a physical barrier that obstructs chemical reactions and molecular diffusion [57]. Numerous methods of microencapsulation have been developed, including centrifugal extrusion, freeze drying, extrusion, and spray drying. Freeze drying is a commonly used technique, particularly for the more costly and sensitive bioactive substances. Freeze drying works at a significantly lower temperature and without oxygen, in contrast to spray drying. A previous study performed by Śliwka et al. [58] employed encapsulation of phages using extrusion-ionic gelation, then drying alginate microspheres using lyophilization process with the addition of 0.3 M mannitol to reduce dehydration. It was found that the highest number of bacteriophages were recovered from encapsulated and freeze-dried microspheres. Another research carried out by Cortés et al. [59] described two different encapsulation methods for bacteriophages using two biocompatible materials: a lipid cationic mixture and an alginate mixture combined with the antacid CaCO_3_. These methods presented effective ways to improve the stability and control the delivery of bacteriophages, which is of tremendous and novel significance in bacteriophage therapy, as it protects the bacteriophages from the challenging environmental conditions. In our study, microencapsulation of *Salmonella* phages using freeze drying with whey protein and trehalose was selected for preparing our formula as recommended by Picot and Lacroix [36] to examine the in vivo impact of the lyophilized isolated phages against MDR-*Salmonella* infected mice via oral infection model [38]. The antibacterial potential of the freeze-dried phage cocktail was evaluated in vitro using the spot test to ensure that the lyophilization process did not negatively impact its activity. That was followed by an in vivo examination of the microencapsulated phages. Many studies demonstrated that the lytic activity of the phage cocktails could effectively suppress bacterial pathogens [60–62]. A study reported that the microencapsulated phages were efficiently released into the simulated intestinal fluid, leading to a better therapeutic effect in rats infected with *E. coli* O157:H7 compared to the effects of the free phages [60]. Another study found that microencapsulated phages exhibited significantly improved stability, gastric acid resistance, and efficacy where they could be potentially applied as biological control agents against *Salmonella* infections [61].

It was also reported that a cocktail of three lytic phages was proven to successfully inhibit *E. coli* [62]. Another study revealed the successful application of two types of anti-*Pseudomonas* phage cocktail (formulated in 35% ethanol in water containing non-ionic polymers) as a novel innovative formula for topical delivery using a metered-dose spray [63]. In our study, it was demonstrated that the freeze-dried formula proved to be an effective mean of delivering the bacteriophage cocktail containing VB_ST_E15 and VB_ST_SPNIS2 over an extended period of time. That was evidenced by the positive spot test that was performed for the released phages from the microcapsules during the course of the in vivo study (about two months). This indicates that the current bacteriophage cocktail could present a promising anti-bacterial candidate against MDR *Salmonella* spp.

The current study demonstrated that bacteriophages treated groups with either the free phage lysate cocktail or the microencapsulated form of the phage cocktail showed mild dilation of hepatic sinusoids in all treated groups, some revealing adenoid pattern, especially in the groups treated with the free form of the phage. Activation of Ito cells could also be detected in phages treated groups. Spleen cells showed a significant number of tingible bodies. MLN revealed development of secondary follicles in treated groups, with obvious germinal centers. Noticeable tingible body macrophages were also demonstrated. This indicates that the treatment with the bacteriophage cocktail successfully induced an immune response against the systemic dissemination of *Salmonella* infections. Furthermore, the reduction in the bacterial count as well as the body weight gain in phages treated groups revealed that phage treatment successfully enhanced the clearance of the *Salmonella* infection. That was in agreement with another study that demonstrated the effectiveness of bacteriophage in the management of salmonellosis caused by *Salmonella* enteritidis in mice models [64]. Moreover, several recent studies have been conducted to confirm the promising lytic activities of the isolated bacteriophages against various clinically relevant pathogens and their potential use in the control of MDR Gram-positive and Gram-negative-associated infections [64, 65]. To the best of our knowledge, this is the first report that demonstrated the broad-spectrum effectiveness of the currently isolated phage cocktail against various MDR *Salmonella* serovars.

## Conclusions

Two lytic phages belonging to the Caudoviricetes class, named VB_ST_E15 and VB_ST_SPNIS2, were isolated from chicken rinse samples, refined, characterized, and evaluated in the current study. Genomic analysis showed that the two phages belonged to the class Caudoviricetes, genus Uetakevirus. The phage cocktail showed excellent thermal and pH stability and maintained its activity for up to 90 days and displayed promising in vitro lytic activity against MDR clinically isolated *Salmonella* serovars. An in vivo study using an oral infection mouse model showed that the isolated phage cocktail, which was prepared as a freeze-dried powder for oral administration, effectively controlled the infection with various MDR *Salmonella* serovars. It also effectively stimulated an immune response against *Salmonella* infections as evidenced by the in vivo experiment. It is therefore clear that the currently isolated and formulated phage cocktail could present a promising oral formula for potential clinical application to combat MDR *Salmonella* infections.

**List of Figure legends**.

### Electronic supplementary material

Below is the link to the electronic supplementary material.

Supplementary Material 1

## Data Availability

No datasets were generated or analysed during the current study.

## Abbreviations

MDR: Multidrug-resistant
ATCC: American Type Culture Collection
NTS: Non Typhoidal *Salmonella*
iNTS: invasive Non Typhoidal *Salmonella*
SSA: Sub-Saharan Africa
BSI: Blood stream infection
AHRI: Animal Health Research Institute
WHO: World Health Organization
TSB: Tryptic Soy Broth
DAO: Double Agar Overlay
SM: Saline Magnesium
TEM: Transmission Electron Microscope
WPI: Whey Protein Isolate
PSI: Pounds per Square Inch
EE: Encapsulation Efficiency
TP: Total Phage titer
RP: Recovered dried Powder
MLN: Mesenteric Lymph Nodes
AMR: Antimicrobial resistance
