# Supplementary material for: Genomic characterization, in vitro, and preclinical evaluation of two microencapsulated lytic phages VB_ST_E15 and VB_ST_SPNIS2 against clinical multidrug-resistant Salmonella serovars
Source: Ann Clin Microbiol Antimicrob. 2024 Feb 15;23:17. doi: 10.1186/s12941-024-00678-3 (PMC10870556; doi:10.1186/s12941-024-00678-3)
Supplement: Supplementary file 1 — Supplementary Material 1 [file 12941_2024_678_MOESM1_ESM.docx]

**Table S1.** Normality tests for data from different groups of mice

| **Variables** | **Description** | **Kolmogorov-Smirnov** | | | **Shapiro-Wilk** | | |
| --- | --- | --- | --- | --- | --- | --- | --- |
|  |  | **Statistic** | **df** | **Sig.** | **Statistic** | **df** | **Sig.** |
| Log bacterial count | infected, treated by free-form | 0.203 | 9 | 0.200 | 0.904 | 9 | 0.278 |
|  | infected, treated by a microencapsulated form | 0.196 | 9 | 0.200 | 0.925 | 9 | 0.436 |
|  | infected, treated by vehicle | 0.152 | 9 | 0.200 | 0.960 | 9 | 0.793 |
|  | infected, untreated | 0.194 | 9 | 0.200 | 0.894 | 9 | 0.222 |
|  | uninfected, untreated | 0.000 | 3 | 0.000 | 0.000 | 3 | 0.000 |
| Weight gain | infected, treated by free-form | 0.149 | 9 | 0.200 | 0.945 | 9 | 0.634 |
|  | infected, treated by a microencapsulated form | 0.180 | 9 | 0.200 | 0.956 | 9 | 0.761 |
|  | infected, treated by vehicle | 0.329 | 9 | 0.006 | 0.809 | 9 | 0.026 |
|  | infected, untreated | 0.185 | 9 | 0.200 | 0.920 | 9 | 0.396 |
|  | uninfected, untreated | 0.213 | 3 | 0.000 | 0.990 | 3 | 0.809 |

The normality of the data was assessed using the Kolmogorov-Smirnov and Shapiro-Wilk tests. Results, in Table S1, showed that the log bacterial count data followed a normal distribution for all groups (Figure S1). The weight gain data (Figure S2) were normally distributed for all groups except the infected, treated by vehicle group, which revealed a substantial deviation from normality (p < 0.05).

**Table S2.** Levene’s test for homogeneity of variances

|  | | **Levine’s test** | **df1** | **df2** | **Sig.** |
| --- | --- | --- | --- | --- | --- |
| **Log bacterial count** | Based on Mean | 1.805 | 4 | 34 | 0.151 |
|  | Based on Median | 1.135 | 4 | 34 | 0.357 |
|  | Based on Median and with adjusted df | 1.135 | 4 | 29.117 | 0.359 |
|  | Based on trimmed mean | 1.766 | 4 | 34 | 0.158 |

The level of significance *p-value* was set at 0.05. The homogeneity of variances assumption was tested using **Levene’s test**. Results in Table S2 revealed that there were no significant differences in the variances of the log bacterial count among the five groups, regardless of the method employed for Levene's test (p > 0.05), Sig, significance..

**Table S3.** Analysis of variance (ANOVA)

|  | | **Sum of Squares** | **df** | **Mean Square** | **F** | **Sig.** |
| --- | --- | --- | --- | --- | --- | --- |
| Log bacterial count | Between Groups | 40.102 | 4 | 10.025 | 145.946 | 0.000 |
|  | Within Groups | 2.336 | 34 | 0.069 |  |  |
|  | Total | 42.437 | 38 |  |  |  |
| Weight gain | Between Groups | 37728.986 | 4 | 9432.246 | 99.993 | 0.000 |
|  | Within Groups | 3207.198 | 34 | 94.329 |  |  |
|  | Total | 40936.184 | 38 |  |  |  |

The level of significance *p-value* was set at 0.05. Analysis of variance. A one-way ANOVA was performed to investigate the effects of different interventions on log bacterial count and mice weight. The findings are presented in Table S3. The findings revealed a statistically significant disparity in the logarithmic bacterial count and mice weight among at least two treatment groups (p < 0.001). These findings indicate that the treatments had distinct impacts on both the bacterial infection and the mice weight. Sig, significance.

**Table S4**. Genomic annotation analysis and the resulting open reading frames (ORFs) of the phage vB_ST_E15. (<https://www.ncbi.nlm.nih.gov/nuccore/OR757455>)

| **ORF** | **Start** | **Stop** | **Strand** | **Putative Function** |
| --- | --- | --- | --- | --- |
| 1 | 2 | 676 | + | Phage terminase, small subunit |
| 2 | 822 | 2297 | + | Phage terminase, large subunit |
| 3 | 2732 | 2340 | - | Hypothetical protein |
| 4 | 3454 | 5124 | + | Phage tail protein |
| 5 | 5480 | 6136 | + | Phage peptidase |
| 6 | 6147 | 7154 | + | Major phage capsid protein [Enterobacteriaceae] |
| 7 | 7167 | 7559 | + | Phage Hypothetical Protein |
| 8 | 8236 | 8841 | + | Phage Hypothetical Protein |
| 9 | 8841 | 11318 | + | Phage Hypothetical Protein |
| 10 | 11318 | 11782 | + | Phage Hypothetical Protein |
| 11 | 11782 | 12324 | + | Phage Hypothetical Protein |
| 12 | 13409 | 14866 | + | Phage Hypothetical Protein |
| 13 | 15200 | 16771 | + | Phage Hypothetical Protein |
| 14 | 20230 | 23442 | + | Right-handed parallel beta-helix repeat-containing protein |
| 15 | 24662 | 23484 | - | O-antigen ligase family protein |
| 16 | 25037 | 25441 | + | Phage holin family protein |
| 17 | 25811 | 26833 | + | Putative Phage glycoside hydrolase |
| 18 | 27470 | 28402 | + | Phage tyrosine-type recombinase/integrase |
| 19 | 29441 | 28863 | - | Putative Phage methyltransferase |
| 20 | 29982 | 29608 | - | Putative transcriptional activator |
| 21 | 31345 | 30323 | - | Phage recombination protein RecT |
| 22 | 32251 | 31355 | - | Phage endonuclease/ recombinase protein |
| 23 | 32574 | 32251 | - | Phage hypothetical protein |
| 24 | 33522 | 32926 | - | Phage helix-turn-helix transcriptional regulator |
| 25 | 34370 | 35365 | + | Phage protein |
| 26 | 35414 | 36091 | + | DNA T-like ssDNA-binding domain-containing protein |
| 27 | 36222 | 36686 | + | Crossover junction endodeoxyribonuclease RuvC |
| 28 | 36743 | 37438 | + | Eae-like protein [Salmonella phage epsilon15] |
| 29 | 37435 | 38268 | + | ead/Ea22-like family protein |
| 30 | 38270 | 38488 | + | Unnamed protein product |
| 31 | 38492 | 39247 | + | DUF551 domain-containing protein |
| 32 | 39512 | 39850 | + | Hypothetical protein |

**Table S5**. Genomic annotation analysis and the resulting open reading frames (ORFs) of the phage VB_ST_SPNIS2. ( [https://www.ncbi.nlm.nih.gov/nuccore/OR757456](https://www.ncbi.nlm.nih.gov/nuccore/OR757455))

| **ORF** | **Start** | **Stop** | **Strand** | **Function** |
| --- | --- | --- | --- | --- |
| 1 | 1 | 594 | + | Terminase small subunit |
| 2 | 591 | 2072 | + | Terminase [Salmonella enterica] |
| 3 | 2611 | 2853 | + | Hypothetical protein |
| 4 | 3308 | 4978 | + | Phage Tail Protein |
| 5 | 5277 | 5987 | + | Peptidase [Citrobacter freundii] |
| 6 | 5998 | 7005 | + | Phage capsid protein |
| 7 | 7018 | 7410 | + | Hypothetical protein |
| 8 | 8087 | 8692 | + | Hypothetical protein |
| 9 | 8692 | 11169 | + | Hypothetical protein |
| 10 | 11633 | 12175 | + | Hypothetical protein [Enterobacteriaceae] |
| 11 | 12961 | 15018 | + | Phage Structural Protein |
| 12 | 15015 | 16817 | + | Hypothetical protein |
| 13 | 16822 | 19296 | + | Hypothetical protein |
| 14 | 19541 | 19834 | + | Hypothetical protein |
| 15 | 19957 | 20340 | + | Tail spike protein |
| 16 | 20550 | 22280 | + | Tail spike protein |
| 17 | 23140 | 22316 | - | Acyltransferase |
| 18 | 23565 | 23188 | - | Acyltransferase [Salmonella enterica] |
| 19 | 24182 | 23571 | - | Acyltransferase [Salmonella enterica] |
| 20 | 24778 | 25182 | + | Putative Phage holin Protein |
| 21 | 25552 | 26178 | + | Glycoside hydrolase family 19 protein [Salmonella enterica] |
| 22 | 27211 | 28146 | + | Tyrosine-type recombinase/integrase |
| 23 | 28955 | 28143 | - | Adenine methylase |
| 24 | 30019 | 28952 | - | DGQHR domain-containing protein |
| 25 | 30541 | 30167 | - | Putative transcriptional activator |
| 26 | 31759 | 30878 | - | RecT family recombinase |
| 27 | 32179 | 31769 | - | Endonuclease |
| 28 | 32984 | 32160 | - | Exodeoxyribonuclease VIII |
| 29 | 33304 | 32981 | - | HP |
| 30 | 34252 | 33656 | - | Helix-turn-helix transcriptional regulator |
| 31 | 35092 | 35847 | + | Pyocin large subunit |
| 32 | 36731 | 37003 | + | Hypothetical protein |
| 33 | 37015 | 37638 | + | Phage Protein |
| 34 | 37889 | 38248 | + | eaf [Salmonella phage P22] Family of unknown function (DUF5448); pfam17526" |
| 35 | 38390 | 38698 | + | Hypothetical protein |


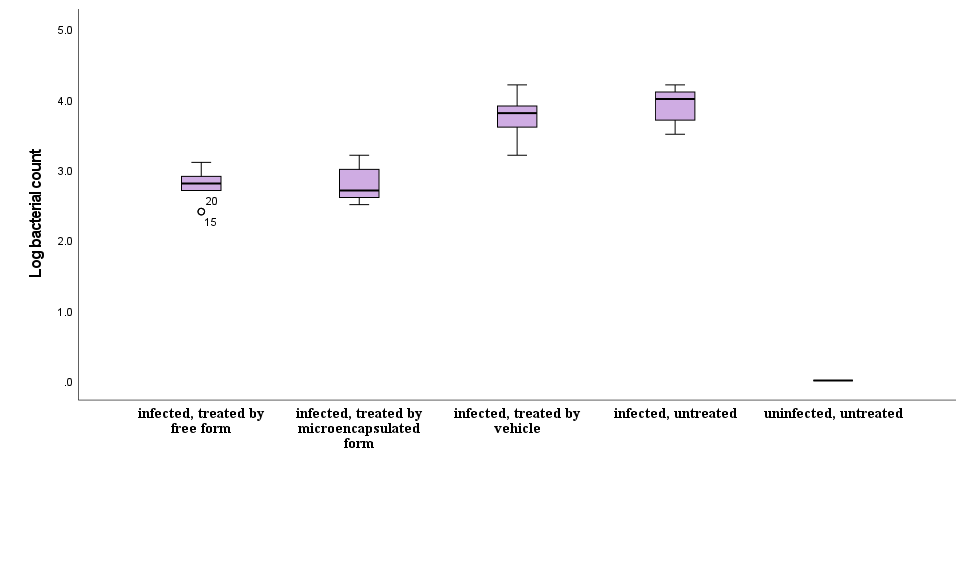


**Figure S1.** **Log bacterial count of different groups of mice under various conditions.** The y-axis represents the log bacterial count, and the x-axis represents the different groups: infected, treated by free form (FF); infected, treated by microencapsulated form (MF); infected, treated by vehicle (V); infected, untreated (I); uninfected, untreated (U). The pink boxes indicate the data distribution.


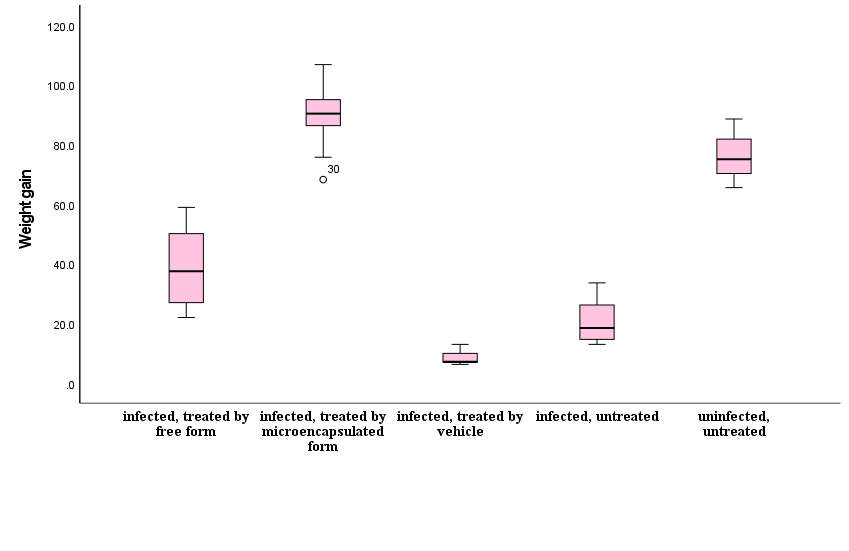


**Figure S2.** **Weight gain of different groups of mice under various conditions.** The y-axis represents the weight gain, and the x-axis represents the different groups: infected, treated by free form (FF); infected, treated by microencapsulated form (MF); infected, treated by vehicle (V); infected, untreated (I); uninfected, untreated (U). The pink boxes indicate the data distribution, and the circles indicate the outliers.
